# Oxidation of p62 as an evolutionary adaptation to promote autophagy in stress conditions

**DOI:** 10.15698/cst2018.04.132

**Published:** 2018-03-23

**Authors:** Elsje G. Otten, Rhoda Stefanatos, Bernadette Carroll, Viktor I. Korolchuk

**Affiliations:** 1 Institute for Cell and Molecular Biosciences (ICaMB); Newcastle University Institute for Ageing (NUIA); Newcastle University; Campus for Ageing and Vitality; Newcastle upon Tyne, UK.

**Keywords:** Amyotrophic Lateral Sclerosis, reactive oxygen species

## Abstract

Ageing and age-related diseases are characterised by increased oxidative and proteotoxic stress, which results in negative effects on cell function and survival. The cell possesses several mechanisms to deal with damaged proteins, including degradation via macroautophagy (hereafter called autophagy). This essential cellular pathway is conserved from yeast to humans and it is well established that its impairment reduces lifespan in multiple model organisms, including worms, flies and mice. In our study, recently published in Nature Communications, we asked if longer lifespan characteristic of higher organisms is the result of evolutionary adaptations to the autophagy machinery. We found that the autophagy receptor p62 can be oxidised leading to its oligomerisation which ultimately promotes autophagy. However this mechanism, present in vertebrates, has been acquired late in evolution. We propose that the ability of p62 to sense reactive oxygen species (ROS) via oxidation, and potentially other similar modifications, may have evolved in higher organisms and contributed to their increased lifespan. Indeed, impairment of this process could result in age-related neurodegeneration in humans.

Autophagy (literally meaning self-eating) is an intracellular trafficking pathway. It involves the formation of a double membraned organelle (called the autophagosome) around components of the cell, such as damaged proteins, protein aggregates and organelles, designated for degradation. Autophagosomes eventually fuse with a lysosome where its cargo is degraded. p62, also known as SQSTM1, is an autophagy receptor protein which is essential for the formation of ubiquitinated protein aggregates and links ubiquitinated cargo to the nascent autophagosome by binding to proteins of the LC3-family. Previous studies have demonstrated that p62 is tightly controlled through post-translational modifications, including ubiquitination and phosphorylation. We have now shown that the activity of p62 is also controlled by oxidation. Specifically, we identified a mechanism by which autophagy can be induced in conditions of oxidative stress via the oxidation-dependent oligomerisation of p62. Oxidised oligomers of p62 are formed by disulphide bonds and appear not only to be serving to recruit cargo to the pre-existing nascent autophagosome but also promote biogenesis of autophagosomes. This function of p62 is essential for the survival of mammalian cells in oxidative stress conditions. This finding adds a new dimension to the regulation of autophagy and has important implications for human ageing and disease where redox balance is perturbed (**Figure 1**).

**Figure 1:**
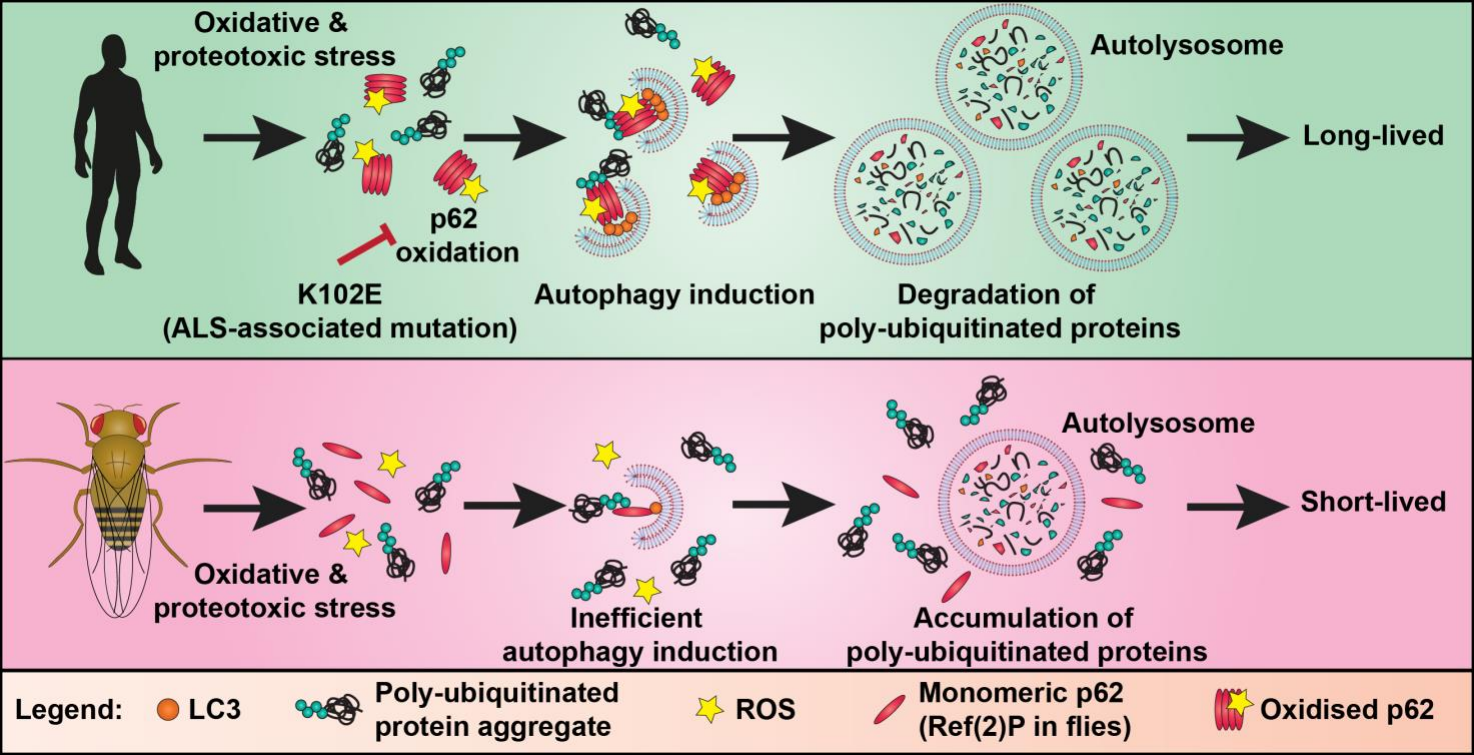
FIGURE 1: Diagram of the proposed evolutionary adaptation to the autophagy machinery involving oxidation of p62. Oxidative and proteotoxic stress triggers oxidation and oligomerisation of p62, promoting the formation of autophagosomes around poly-ubiquitinated proteins. After the autophagosome is fully formed it fuses with a lysosome to form the autolysosome. In the autolysosome, the cargo (e.g. poly-ubiquitinated protein aggregates and p62) is degraded by lysosomal proteases. The ALS-associated mutation K102E can impair oxidation of p62, resulting in less autophagy induction in oxidative and proteotoxic stress conditions. The key cysteine residues required for p62 oxidation, present in vertebrates, were acquired late in evolution. Ref(2)P, the fly homolog of p62, lacks these oxidation-sensitive cysteine residues. Introduction of the p62 redox-sensitive cysteines into Ref(2)P promotes autophagy and survival in oxidative and proteotoxic stress conditions. This suggests that longevity associated with vertebrates, like humans, is a result of evolutionary adaptations to their autophagy machinery, which allow them to better manage the age-related increase in proteotoxic burden.

We identified two cysteines (Cys105 and Cys113) in p62, located within the flexible linker region between the PB1 and ZZ domains, which can sense the saturation of cellular antioxidant systems. Mutation of these residues abolished the ability of p62 to form oxidised oligomers and ubiquitinated aggregates in oxidative stress conditions. As a result, the ROS-insensitive mutant was unable to support p62-dependent induction of autophagy and the degradation of poly-ubiquitinated proteins in oxidative stress conditions.

Phylogenetic analysis revealed that these key cysteine residues are only present in vertebrates, suggesting that the ability of p62 to promote autophagy in response to oxidative stress has been acquired late in evolution. Consistent with these cysteines being integral for p62 redox sensing, the fly homologue of p62, called Ref(2)P, has a similar domain structure to p62, but the protein showed no evidence of oxidation-induced oligomerisation. This offered us a unique opportunity to test whether the introduction of redox-sensitive residues into Ref(2)P would promote autophagy and mediate increased stress resistance in the fly. We used CRISPR/Cas9 genome editing to ‘humanise’ Ref(2)P by introducing the redox-regulated cysteines of p62. Indeed, flies expressing oxidisable Ref(2)P displayed higher levels of basal autophagy and an increased resistance to oxidative and thermal stress (**Figure 1**). Despite that "humanised" flies were able to survive for longer under stress with age, we did not observe an increase in longevity in basal conditions. We speculate that this is related to the poor efficiency of the autophagy pathway as a whole after a certain age in flies. Our data support the hypothesis that longer-lived species, like vertebrates, have evolved adaptations to their autophagy machinery, which allow management of the increased proteotoxic burden, which comes with age.

Our data shows that acquirement of redox regulation of the autophagy machinery improves stress resistance, but what are the consequences if this redox regulation is impaired? We wondered if perturbation of p62 oxidation could underlie age-associated disease. Amyotrophic Lateral Sclerosis (ALS) is one such late-adult onset neurodegenerative diseases characterised by the progressive degeneration of motor neurons. We became interested in a sporadic ALS case carrying a mutation K102E because it is located in the same linker region as the redox-regulated cysteines we had identified. Introduction of this mutation into human p62 impaired the formation of oxidised oligomers. Moreover, it abolished the ability of p62 to promote the induction of protein aggregates and autophagosomes (**Figure 1**). These findings support the hypothesis that perturbation of p62 oxidation could contribute to age-associated neurodegeneration. It would be important to validate these conclusions using animal models and patient tissue.

Our published observations are one of the first examples of redox-regulation of receptor-mediated selective autophagy. Most of our experiments were performed in oxidative stress conditions, but we observed that in basal and starvation conditions a small proportion of p62 protein was oxidised. Furthermore, in starvation conditions, even in the absence of exogenous ROS, redox-sensitive cysteine residues of p62 were also important for autophagy induction. It has been previously shown that starvation induces ROS production. It would be interesting to test if localised ROS production in basal or starvation conditions could contribute to the induction of autophagy via oxidation of p62.

How p62 can be specifically and efficiently oxidised by H_2_O_2 _remains unknown. Recently however, it has become clear that it is unlikely that redox-regulated proteins, such as p62, are directly oxidised by ROS such as H_2_O_2. _When ROS are produced, they primarily react with the family of peroxiredoxin proteins, which contain highly reactive cysteines. Interestingly, peroxiredoxins have been shown to function as redox-relay proteins by transferring oxidant equivalents to target proteins. It could be hypothesised that a local ROS signal, via a peroxiredoxin, could oxidise p62 resulting in its oligomerisation and the initiation of autophagy. Consistent with this hypothesis, multiple peroxiredoxins have been found on autophagosomal membranes and our unpublished data suggest that peroxiredoxins indeed associate with disulphide-mediated p62 oligomers.

Our study focused on the degradation of poly-ubiquitinated proteins in stress conditions. However, it would also be of interest to test if other selective autophagy pathways could be initiated by the oxidation of p62 or whether the function of other autophagy receptors is similarly regulated by oxidation-dependent oligomerisation. Our findings may be the first example of a general mechanism facilitating the induction of autophagosome formation at the site where it is needed most. For example, damaged mitochondria could release ROS and promote localised oxidation of receptors, resulting in the recruitment of the autophagy machinery and degradation of the mitochondria via mitophagy. It is an interesting idea that the requirement for the formation of autophagosomes can be coordinated by such mechanisms with the irreversible damage to intracellular components requiring their removal.

In conclusion, our data suggest that redox-regulation of p62 promotes autophagy in stress conditions, thereby helping to maintain cellular homeostasis. Stimulating disulphide-mediated oligomerisation of p62 and other autophagy receptors could be a promising therapeutic target, potentially allowing targeted activation of receptor-dependent autophagy pathways. This would be relevant for a wide range of diseases where autophagy has been implicated, including neurodegeneration and cancer.

